# Effect of time delay in inter-hospital transfer on outcomes of endovascular treatment of acute ischemic stroke

**DOI:** 10.3389/fneur.2023.1303061

**Published:** 2023-12-22

**Authors:** Keshet Pardo, Jonathan Naftali, Rani Barnea, Michael Findler, Alain Perlow, Ran Brauner, Eitan Auriel, Guy Raphaeli

**Affiliations:** ^1^Department of Neurology, Rabin Medical Center – Beilinson Hospital, Petah Tikva, Israel; ^2^Sackler School of Medicine, Tel Aviv University, Tel-Aviv, Israel; ^3^Department of Radiology, Rabin Medical Center – Beilinson Hospital, Petah Tikva, Israel

**Keywords:** endovascular treatment, ischemic stroke, thrombectomy, patient transfer, time to treatment

## Abstract

**Background:**

Endovascular treatment (EVT) with mechanical thrombectomy is the standard of care for large vessel occlusion (LVO) in acute ischemic stroke (AIS). The most common approach today is to perform EVT in a comprehensive stroke center (CSC) and transfer relevant patients for EVT from a primary stroke center (PSC). Rapid and efficient treatment of LVO is a key factor in achieving a good clinical outcome.

**Methods:**

We present our retrospective cohort of patients who underwent EVT between 2018 and 2021, including direct admissions and patients transferred from PSC. Primary endpoints were time intervals (door-to-puncture, onset-to-puncture, door-to-door) and favorable outcome (mRS ≤ 2) at 90 days. Secondary outcomes were successful recanalization, mortality rate, and symptomatic intracranial hemorrhage (sICH). Additional analysis was performed for transferred patients not treated with EVT; endpoints were time intervals, favorable outcomes, and reason for exclusion of EVT.

**Results:**

Among a total of 405 patients, 272 were admitted directly to our EVT center and 133 were transferred; there was no significant difference between groups in the occluded vascular territory, baseline NIHSS, wake-up strokes, or thrombolysis rate. Directly admitted patients had a shorter door-to-puncture time than transferred patients (190 min vs. 293 min, *p* < 0.001). The median door-to-door shift time was 204 min. We found no significant difference in functional independence, successful recanalization rates, or sICH rates. The most common reason to exclude transferred patients from EVT was clinical or angiographic improvement (55.6% of patients).

**Conclusion:**

Our results show that transferring patients to the EVT center does not affect clinical outcomes, despite the expected delay in EVT. Reassessment of patients upon arrival at the CSC is crucial, and patient selection should be done based on both time and tissue window.

## Introduction

Endovascular treatment (EVT) with mechanical thrombectomy is the standard of care for acute ischemic stroke (AIS) patients with proximal large vessel occlusion (LVO) (1–3). Rapid reperfusion is critical in order to achieve favorable clinical outcomes (4).

EVT is not accessible in primary stroke centers (PSC); therefore, selected EVT candidates are transferred from these centers (5). The common practice of managing these patients is to administer intravenous thrombolysis (IVT) at the PSC and later transport them to a comprehensive stroke center (CSC), equipped with EVT capabilities (“drip-and-ship”) (5–7).

Patient transfer leads to a delay in onset-to-puncture time (8). Previous studies have shown that transferred patients suffer from worse functional outcomes (modified Rankin Scale [mRS] > 3) (9, 10), and higher mortality rates compared to directly admitted patients (11). Moreover, prolonged transfer time results in the exclusion of patients from EVT (12, 13). There is an ongoing debate about whether the decision to administer EVT should be based on a time window or tissue window (e.g., CT findings, ASPECT score, CT perfusion) (14–17).

In Israel, there are 9 CSCs and another 16 PSCs that provide IVT only. Geographically, the distribution of CSCs is uneven, causing populations from the periphery to be significantly delayed on arrival to a CSC after AIS onset.

Our aim in this study was to assess the delay in EVT among transferred patients and its effect on clinical and procedural outcomes. In addition, we analyzed radiological and clinical differences in patients who were transferred but did not undergo EVT, and the reasons for EVT exclusion. These findings could improve patient selection when considering transfer to CSC.

## Methods

### Study design and population

We conducted a single-center retrospective analysis of all AIS patients due to LVO of the anterior or posterior circulation who underwent EVT between 2018 and 2021 at Rabin Medical Center, Israel.

Our cohort was dichotomized into a group of directly admitted patients (DAG) and a group of transferred patients (TG) who arrived first at a PSC. Additional data was collected on patients with AIS due to LVO who were transferred to our center for EVT but were found ineligible after re-evaluation.

All transferred patients were clinically re-evaluated using the National Institutes of Health Stroke Scale [NIHSS (18)]. Selected patients, specifically those with prolonged door-to-door time (over 1 h) or clinical worsening, were referred to a repeat stroke imaging protocol that included CT, CTA, and CTP, followed by reconsideration of eligibility for EVT. An experienced stroke neurologist reviewed patient records to determine the reason for exclusion from EVT and classified them as one of the following: (1) Clinical improvement (i.e., repeated NIHSS ≤4); (2) Imaging improvement (i.e., recanalization on repeated CTA); (3) Clinical worsening; (4) Imaging worsening (i.e., new ASPECT score less than 5 on repeat NCCT); (5) Patient refusal of EVT.

The functional outcome of the mRS at 90 days after a stroke was consistently recorded either by a follow-up visit at a post-stroke outpatient clinic or virtually by telephone.

Patients with in-hospital acute stroke, occurring while hospitalized for reasons other than ischemic stroke, were excluded. We also excluded patients who were transferred for observation, pending a decision regarding EVT based on clinical worsening.

The study was approved by the local ethical committee. Due to the retrospective, non-interventional design of this work, informed consent was not required.

The study followed the guidelines for observational cohorts according to Strengthening the Reporting of Observational Studies in Epidemiology (STROBE) (19).

### Outcome endpoints

The primary endpoints were time intervals: (1) Onset-to-CSC (OTC) – the time from onset of symptoms to arrival at CSC. (2) Onset-to-puncture (OTP) – the time from symptom onset to arterial puncture at CSC. (3) Door-to-puncture (DTP) – the time from arrival at the first medical center (i.e., CSC for directly admitted patients and PSC for transferred patients) to arterial puncture of EVT. (4) Door-to-door (DTD) shift – the time from arrival at PSC to CSC; and (5) Functional independence at day 90, defined as mRS ≤ 2.

The secondary endpoints were successful recanalization using thrombolysis in cerebral infarction (TICI), intracranial hemorrhage (ICH), both asymptomatic and symptomatic (defined as any CT-documented hemorrhage, with temporal relation to clinical deterioration), and mortality rate.

A secondary analysis was performed on patients who were transferred and excluded from EVT with respect to time intervals (OTC and DTD shift), functional independence at day 90, and reasons for not performing EVT.

### Statistical analysis

Statistical analysis was performed using IBM SPSS Statistics for Windows, version 25.0 (IBM Corp., Armonk, NY). Qualitative data were presented as frequencies and percentages. Pearson’s chi-squared test was used for comparisons. Quantitative data were presented as the median (IQR) for non-normally distributed data and as the mean ± SD for normally distributed data. A T-test was used to compare demographic and time interval data. A P value ≤0.05 was considered statistically significant.

## Results

**Figure 1:**
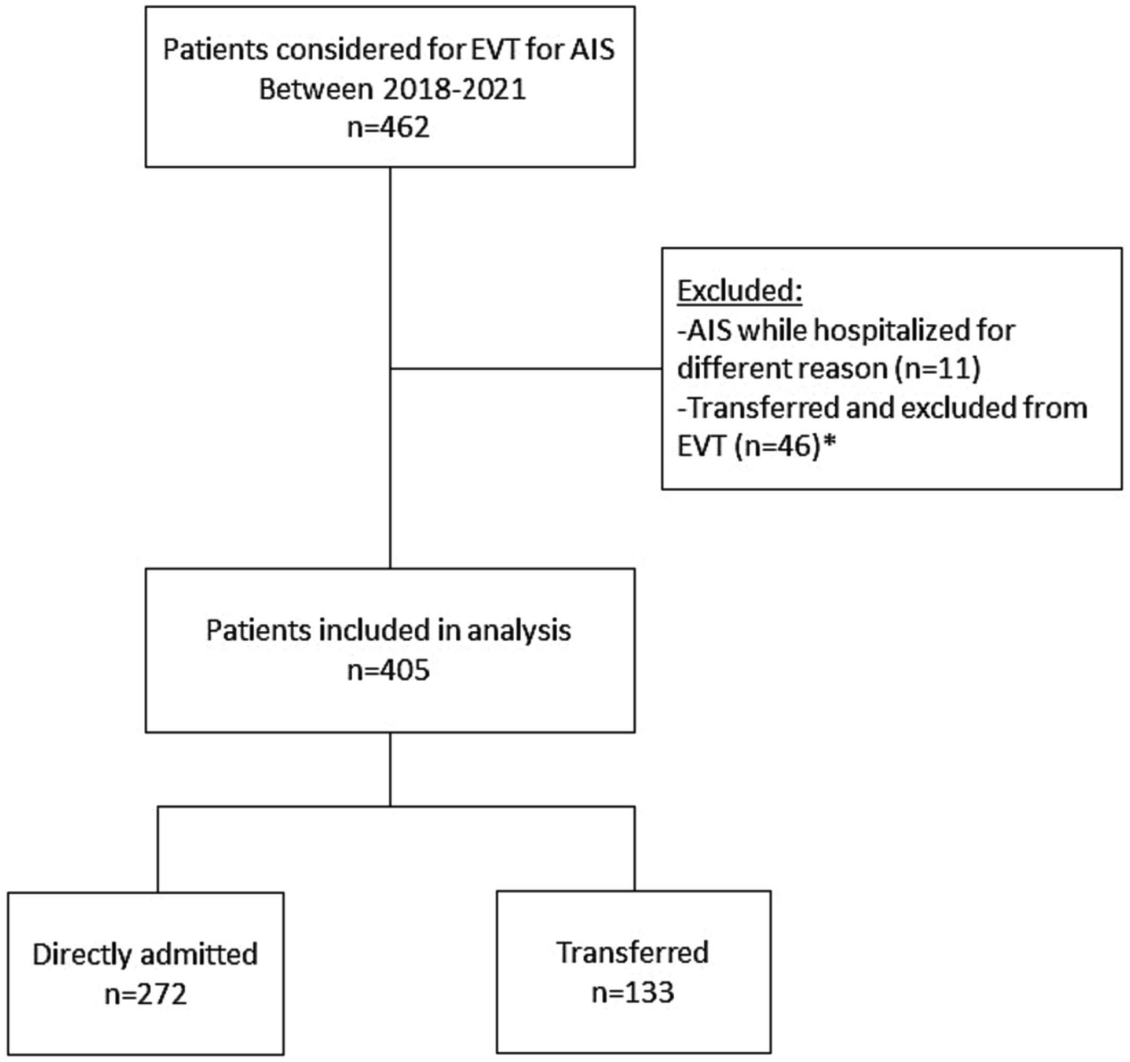
Patient selection flowchart. EVT, Endovascular treatment; AIS, acute ischemic stroke.

Our study included a total of 405 patients who underwent EVT for AIS with LVO between January 2018 and December 2021. Among these patients, 272 (67%) were categorized in the directly admitted group (DAG), and 133 (33%) were in the transferred group (TG) Figure 1.

### Baseline characteristics

There were no demographic differences between the two groups. The median age was 75 years (IQR, 64.3–83.8) and 77 years (IQR, 66.5–83.5) in the DAG and TG groups, respectively. Female patients were 139 (51%) and 66 (50%), respectively. There were no differences between groups in cardiovascular risk factors, previous stroke, transient ischemic attack (TIA) percentages, or NIHSS at presentation – median 14 (IQR, 9–17) and 14 (IQR, 9–17.5) in DAG and TG, respectively. There were no differences between the groups in the vascular territory of the occluded vessel, wake-up stroke, or IVT percentage. Table 1 presents baseline clinical and demographic data.

**Table 1:** Demographics, baseline, and stroke characteristics.

	Directly admitted *n* = 272	Transferred *n* = 133	*p* value
Female subjects, *n* (%)	139 (51.1)	66 (49.6)	0.780
Age, median (IQR)	75 (64.3–83.8)	77 (66.5–83.5)	0.527
NIHSS at presentation, median (IQR)	14 (9–17)	14 (9–17.5)	0.762
*Medical history*			
Hypertension, *n* (%)	171 (62.9)	95 (71.4)	0.088
Atrial fibrillation, *n* (%)	86 (31.6)	52 (39.1)	0.136
Diabetes mellitus, *n* (%)	86 (31.6)	49 (36.8)	0.295
Dyslipidemia, *n* (%)	148 (54.4)	64 (48.1)	0.234
History of smoking, *n* (%)	42 (15.4)	12 (9)	0.074
Ischemic heart disease, *n* (%)	61 (22.4)	38 (28.6)	0.177
Prior stroke/TIA, *n* (%)	77 (28.3)	27 (20.3)	0.083
Prior CEA, *n* (%)	5 (1.8)	2 (1.5)	0.808
*Stroke characteristics*			
Wake-up stroke, *n* (%)	95 (34.9)	38 (28.8)	0.218
IVT, *n* (%)	98 (36)	41 (31.1)	0.324
Vascular occlusion, *n* (%)			
ICA	72 (26.5)	38 (28.8)	0.949
Tandem occlusion	15 (5.5)	7 (5.3)	
MCA	164 (60.3)	76 (57.6)	
ACA	4 (1.5)	3 (2.3)	
PCA	9 (3.3)	3 (2.3)	
Basilar	20 (7.4)	11 (8.3)	
Vertebral	3 (1.1)	1 (0.8)	

NIHSS, National Institutes of Health Stroke Scale Score; TIA, transient ischemic attack; CEA, carotid endarterectomy; IVT, intravenous thrombolysis.

### Time intervals

The TG had a longer mean OTC time of 404 ± 298 min, compared to 256 ± 287 min for DAG (*p* < 0.001), and a longer mean DTP time of 239 ± 161 min, compared to 190 ± 116 min for DAG (*p* < 0.001). There was no statistically significant difference in OTP time of 478 ± 300 and 428 ± 303 for TG and DAG, respectively.

The DTD interval for TG was 204 ± 154 min. Time intervals are presented in Table 2.

**Table 2:** Time intervals.

Minutes, mean (SD)	Directly admitted *n* = 272	Transferred *n* = 133	*p* value
Onset-to-CSC center	256 (287)	404 (298)	**<0.001**
Door-to-Door		204 (154)	
Door-to-Puncture	190 (116)	293 (161)	**<0.001**
Onset-to-Puncture	428 (303)	478 (300)	0.137

CSC, Comprehensive Stroke Center; PSC, Primary Stroke Center; Door-to-Door, PSC to CSC; Door-to-Puncture, 1st Door (either PSC or CSC) to-Puncture.

### Procedural and clinical outcomes

**Figure 2:**
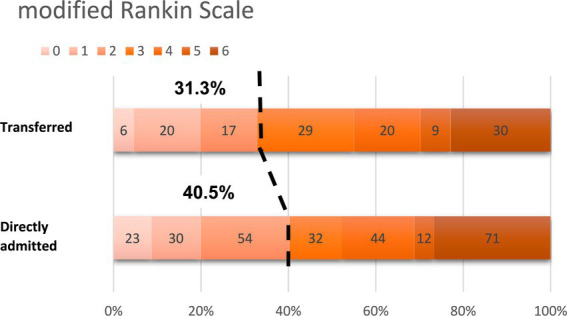
Functional outcome measured as modified Rankin Scale score at 90 days.

There was no statistically significant difference in functional independence (mRS ≤ 2) at day 90, with 41 (31.3%) of the TG compared to 109 (40.5%) of the DAG (*p*  value = 0.074) (data shown in Table 3 and Figure 2).

**Table 3:** Procedural and clinical outcomes.

*n* (%)	Directly admitted *n* = 272	Transferred *n* = 133	*p* value
Successful reperfusion (TICI ≥2B)	236 (88.7)	121 (91.7)	0.363
aICH	27 (9.9)	12 (9)	0.772
sICH	16 (5.9)	11 (8.3)	0.366
Favorable outcome at 90 days (mRS of 0–2)	109 (40.5)	41 (31.3)	0.074
Mortality at 90 days	71 (26.4)	30 (22.9)	0.45

TICI, Thrombolysis in Cerebral Infarction score; ICH, intracranial hemorrhage; aICH, asymptomatic ICH; sICH, symptomatic ICH; mRS, modified Rankin Scale.

There was no difference between the two groups in the rate of successful reperfusion of TICI 2b-3 in 121 (91.7%) of TG and 236 (88.7%) of DAG. Similar rates of ICH were documented in the two groups: 12 (9%) and 27 (9.9%) of asymptomatic ICH and 11 (8.3%) and 16 (5.9%) of symptomatic ICH in TG and DAG, respectively. No difference was found in the mortality rate at 90 days, with 30 (22.9%) cases in TG and 71 (26.4%) in DAG (Table 3).

### Transfers without EVT

Thirty-six patients were transferred and did not undergo EVT. Transferred patients without EVT had similar baseline characteristics compared to transferred patients with EVT, as shown in Supplementary Table 1s.

The incidence of wake-up strokes and IVT was similar in these groups of patients. Transferred patients without EVT had a less severe stroke presentation with a median NIHSS of 6 (IQR; 2.5–12.5) compared to 14 (IQR; 9–17.5) in transferred patients with EVT (p = 0.001) and a higher percentage of LVO involving the posterior circulation (Supplementary Table 1s).

The time intervals of patients without EVT were similar to those with EVT, with an OTC time of 470 ± 384 (mean ± SD) minutes and 404 ± 298 min, respectively, and a DTD time of 254 ± 235 and 204 ± 154 min, respectively (Supplementary Table 2s).

The group transferred without EVT had achieved a more favorable outcome at 90 days (mRS ≤ 2) compared to the group transferred with EVT: 23 patients (63.6%) compared to 41 (31.3%), respectively, *p* < 0.001. There was no statistically significant difference in mortality rates at 90 days between the two groups (Supplementary Table 2s).

The most common reason why EVT was excluded was either clinical or imaging improvement in 20 patients (55.6%), of which 4 patients had only imaging improvement (spontaneous revascularization, without significant clinical change). Fifteen patients (41.7%) had either clinical or imaging worsening and were no longer suitable for EVT, of which only one patient had isolated clinical worsening without supporting imaging findings. One patient refused EVT on arrival (Supplementary Figure 1s).

## Discussion

In the present study, we found that the time delay caused by inter-hospital transfers in patients with LVO who were candidates for EVT did not affect clinical outcomes.

Transferred patients were noted to have longer arrival times at a CSC and longer DTP times. However, OTP time was not found to be longer, suggesting that the decision-making process for EVT was relatively fast and efficient upon arrival at the CSC.

Previous studies have demonstrated that delaying EVT in transferred patients results in worse clinical outcomes (3, 9, 11, 20–22), suggesting that direct transfer to a CSC is more beneficial than the “drip-and-ship” strategy, even at the expense of delayed IVT (23, 24). A large meta-analysis on the subject also favored the direct admission approach (25) (Supplementary Table 3s lists the different studies comparing the clinical outcomes of directly admitted and transferred patients).

The lack of differences between transferred and directly admitted patients found in our study has been described in previous works, although they had limited inclusion criteria to the anterior circulation only and onset-to-puncture time up to 6 h (6, 26) or 12 h (27). A recent randomized control trial also found no differences between directly admitted and transferred AIS patients but included patients with both LVO and non-LVO AIS (28). In contrast, our study included a wider time window and patients presenting with LVO in both the anterior and posterior circulation, which better reflects AIS patients treated with EVT.

There are no clear guidelines on whether transferred patients should undergo repeat imaging prior to EVT. Our practice is to re-evaluate the NIHSS score on arrival and perform a repeat imaging protocol that includes CT, CTA, and CTP in cases of delayed transfer or clinical worsening, followed by a re-evaluation of eligibility for EVT. This selection process is, to our knowledge, the major contributor to the good outcome of transfer patients in our cohort.

Transferred patients who did not receive EVT had lower NIHSS scores and higher rates of posterior circulation stroke. The most common reasons for avoiding EVT were either an improved NIHSS score on arrival or re-canalization on imaging. These subgroups of patients had better clinical outcomes compared to transferred patients who underwent EVT. Indeed, previous studies have suggested that clinical improvement (29) is the most important argument to avoid EVT.

Less than half of our patients were not treated with EVT due to clinical and radiological deterioration, rendering them unsuitable for the procedure based on a worsening ASPECT score (30) or a large ischemic core on CTP (16).

It is important to emphasize that there was no significant difference in OTC and DTD times between transferred patients undergoing EVT and their counterparts not undergoing EVT, suggesting that it was not late arrival that excluded patients from undergoing EVT. This finding is in contrast with previous studies that blamed time delay and deviation from the accepted time window as the primary cause for avoiding EVT or not transferring patients to CSC (12, 27).

Faster transfer times are imperative for good clinical outcomes and are the focus of several studies that predict longer transfer times in the elderly population and emphasize the importance of early communication with the receiving CSC (31, 32). There are different strategies to reduce time intervals in AIS patients, including strategies aimed at improving workflow, having available staff members, considering local anesthesia or conscious sedation (33), and the use of either a countdown clock (34) or a feedback mechanism (35) in order to improve awareness of time. These strategies have been found to be effective in improving both time intervals and clinical outcomes. We believe that crucial contributing factors to the favorable clinical outcome in our cohort were the fast re-evaluation, organization, and response of the medical team in advance of the transfer.

Our study has several limitations. First, because our study is retrospective, some transfer patients not undergoing EVT may be missed; however, we believe that our data provide a good understanding of their clinical considerations regarding EVT. Second, our institution does not have a uniform protocol for repeat imaging on arrival in transferred patients, which is subject to variation among decision-makers. Additional limitations were the small sample size and the unicentric nature of this study.

### Conclusion

There is an ongoing debate as to whether transferring patients to a CSC for EVT is the better approach compared to multiple low-volume thrombectomy units. Our results support the notion that transferring patients to an EVT center does not compromise clinical outcomes, despite the expected delay in EVT. Reassessment of patients upon arrival at the CSC is crucial, and patient selection should be based on both time and tissue window.

## Data availability statement

The raw data supporting the conclusions of this article will be made available by the authors, without undue reservation.

## Ethics statement

The studies involving humans were approved by Rabin medical center REC number: 0703-21-RMC. The studies were conducted in accordance with the local legislation and institutional requirements. Written informed consent for participation was not required from the participants or the participants’ legal guardians/next of kin in accordance with the national legislation and institutional requirements.

## Author contributions

KP: Data curation, Formal analysis, Investigation, Resources, Writing – original draft. JN: Data curation, Writing – original draft. RBa: Data curation, Writing – original draft. MF: Data curation, Writing – original draft. AP: Data curation, Writing – original draft. RBr: Data curation, Writing – original draft. EA: Writing – review & editing. GR: Conceptualization, Data curation, Investigation, Methodology, Writing – review & editing.
