# Supplementary material for: Effect of time delay in inter-hospital transfer on outcomes of endovascular treatment of acute ischemic stroke
Source: Front Neurol. 2023 Dec 22;14:1303061. doi: 10.3389/fneur.2023.1303061 (PMC10766796; doi:10.3389/fneur.2023.1303061)
Supplement: Supplementary file 1 [file Table_1.DOCX]

Supplements:

| **Supplement Table 1s** | | | |
| --- | --- | --- | --- |
| Baseline and stroke characteristics of transferred patients | | | |
|  | **No EVT** n=36 | **EVT** n=133 | **P Value** |
| Female, n (%) | 17 (47.2) | 66 (49.6) | 0.798 |
| Age, median (IQR) | 73 (69-83) | 77 (66.5-83.5) | 0.093 |
| NIHSS at presentation, median (IQR) | 6 (2.5-12.5) | 14 (9-17.5) | **0.001** |
| **Medical history** | | | |
| Hypertension, n (%) | 25 (69.4) | 95 (71.4) | 0.816 |
| Atrial fibrillation, n (%) | 11 (30.6) | 52 (39.1) | 0.347 |
| Diabetes mellitus, n (%) | 13 (36.1) | 49 (36.8) | 0.936 |
| Dyslipidemia, n (%) | 17 (47.2) | 64 (48.1) | 0.924 |
| Ischemic heart disease, n (%) | 11 (30.6) | 38 (28.6) | 0.816 |
| Prior Stoke/TIA, n (%) | 4 (11.1) | 27 (20.3) | 0.206 |
| **Stroke Characteristics** | | | |
| Wake-up stroke, n (%) | 9 (25) | 38 (28.8) | 0.654 |
| IVT, n (%) | 10 (27.8) | 41 (31.1) | 0.704 |
| **Vascular occlusion, n (%)** | | | |
| ICA | 5 (13.9) | 38 (28.8) | **0.011** |
| MCA | 23 (63.9) | 76 (57.6) |  |
| ACA | 0 (0) | 3 (2.3) |  |
| PCA | 2 (5.6) | 3 (2.3) |  |
| Basilar | 2 (5.6) | 11 (8.3) |  |
| Vertebral | 4 (11.1) | 1 (0.8) |  |
| EVT, Endovascular treatment; NIHSS, National Institutes of Health Stroke Scale Score; TIA, transient ischemic attack; CEA, carotid endarterectomy; IVT, intravenous thrombolytic | | | |

| **Supplement - Table 2s** | | | |
| --- | --- | --- | --- |
| **Time intervals** | | | |
| minutes, mean (SD) | **No EVT** n=36 | **EVT** n=133 | **P Value** |
| Onset-EVT center | 470 (384) | 404 (298) | 0.298 |
| Door-Door | 254 (235) | 204 (154) | 0.192 |
| **Clinical outcomes at transferred patients** | | | |
| Num (%) |  |  |  |
| Functional independence at 90 days (mRS of 0–2) | 23 (63.9) | 41 (31.3) | **<0.001** |
| Mortality at 90 days | 5 (13.9) | 30 (22.9) | 0.078 |
| EVT, Endovascular treatment; CSC, Comprehensive stroke center; PSC, primary stroke center; Door-Door, PSC to CSC  mRS, modified Rankin Scale | | | |

| **Supplement - Table 3s** | | | | | | | | | | |
| --- | --- | --- | --- | --- | --- | --- | --- | --- | --- | --- |
| Previous studies comparing directly admitted to transferred patients | | | | | | | | | | |
|  | **n** | **Favourable outcome (mRS of 0–2)** | | **Successful recanalization (TICI ≥2B)** | | **sICH** | | **Mortality** | | Comments |
|  |  | DAG | TG | DAG | TG | DAG | TG | DAG | TG |  |
| Gerschenfeld *et al*., 2017 | 159 | 50.8% | 61.1% | 79.7% | 84.0% | 3.4% | 2.2% | NA | NA | Patient eligible for EVT only within 6 hours after symptom onset |
| K *et al*., 2021 | 282 | ^a^No difference | | 72.7% | 72.6% | NA | NA | ^a^No difference | |  |
| Venema *et al*., 2019  (MR CLEAN) | 1,526 | ***42.4%** | ***33.9%** | 56.6% | 58.7% | 6.4% | 5.4% | 26.4% | 31.5% | Worse functional outcome in transferred patients (adjusted common odds ratio [OR] 0.75 [95% CI, 0.62–0.90] |
| Rinaldo *et al*., 2017 | 8533 | *38.30%* | *33.80%* | 71% | 75.70% | 3.2% | 10.3% | ***10.3%** | ***18.6%** |  |
| Shah *et al*., 2019 | 37,260 | **^c^37.2%** | **^c^33.1%** | NA | NA | ***5.6%** | ***7.0%** | ***13.4%** | ***14.7%** |  |
| Froehler *et al*., 2017 | 984 | ***60.0%** | ***52.2%** | 88.0% | 87.6% | NA | NA | 15.1% | 13.7% | Favourable outcome for directly admitted patients - OR, 1.38; 95% CI, 1.06–1.79; P=0.02 |
| Pérez de la Ossa, N. et al | 1,401 | ^a^No difference | | NA | NA | NA | NA | 27.3% | 27.2% | Radomised control trial, including LVO and non-LVO AIS patients. |
| Brochado, A. P. et al | 375 | 50% | 55% | 88% | 92% | 5% | 3% | 19% | 15% | Patient eligible for EVT only within 6 hours after symptom onset or mismatch on perfusion CT scan |
| Romoli, M. el al | 7,017 | DAG group - higher rates of favorable outcomes. | | ^b^No difference | | ^b^No difference | | ^b^No difference | | **Meta-Analysis** of 18 different studies.  Favourable outcome for directly admitted patients -OR, 1.34; 95% CI, 1.16 to 1.55; I2=30% |
| Our study | 405 | 40.5% | 31.3% | 88.7% | 91.7% | 5.9% | 8.3% | 26.4% | 22.9% | No effect of transfers on higher mRS at 90 days [OR 0.963 (95% CI, 0.664-1.397)] |
| Included studies with comparison of clinical outcomes (mRS at 90 days) after endovascular treatment between directly admitted patients and transferred patients.  Data presented in percentage.  DAG, directly admitted group; TG, Transferred group.  *Statistically significant result, P<0.05  ^a^No difference between groups (no numbers reported)  ^b^No difference between groups – data reported with OR.  ^c^ Reported independent on discharge - OR 0.78 (95% CI, 0.72–0.84) P<0.0001  NA, not available; n, total of patients included in the study | | | | | | | | | | |

**Figure 1s**

EVT, endovascular treatment.

*Imaging worsening includes patients with and without clinical worsening

**Clinical improvement includes patients with and without imaging worsening
